# Intestinal adaptation of crayfish to pond and rice paddy farming: a microbiome-enzyme perspective

**DOI:** 10.3389/fmicb.2026.1989196

**Published:** 2026-10-26

**Authors:** Gao Gao, Jingnan Wei, Lingyu Gan, Huiying Wang, Hongjia Zhang, Xinran Yu, Baoliang Bi

**Affiliations:** 1 College of Animal Science and Technology, Yunnan Agricultural University, Kunming, China; 2 Key Laboratory of Plateau Fishery Resources Protection and Sustainable Utilization in Universities of Yunnan Province, Kunming, Yunnan, China; 3 International College, Yunnan Agricultural University, Kunming, Yunnan, China

**Keywords:** gut content-associated microbiota, intestinal physiology, physiological trade-offs, pond monoculture, *Procambarus clarkii*, rice-crayfish co-culture

## Abstract

The red swamp crayfish (*Procambarus clarkii*) is a globally significant aquaculture species, yet the biological trade-offs between intensive pond monoculture and integrated rice-crayfish co-culture systems remain poorly understood at the intestinal level. This study investigated the intestinal physiological and microbial adaptations of *P. clarkii* reared in pond (high-input formulated feed) and rice paddy (low-input natural diet) systems for 60 days. We integrated analyses of intestinal digestive, antioxidant, immune barrier, and energy metabolism enzymes with 16S rRNA sequencing of the gut content-associated microbiota. Rice paddy-cultured crayfish exhibited significantly higher amylase activity, enhanced antioxidant enzyme activities (SOD, CAT, GSH-Px), and lower oxidative damage (MDA), alongside higher immune barrier enzyme activities (AKP, ACP) and reduced Na^+^/K^+^-ATPase activity compared to pond-cultured crayfish. Conversely, pond-cultured crayfish showed elevated lipase and total protease activities, lower immune barrier enzyme activities, and greater energy transport workload. Microbial analysis of intestinal lumen contents revealed higher lumen-associated microbial diversity in the rice paddy group, with enrichment of Bacteroidota and taxa commonly associated with polysaccharide degradation, whereas the pond group showed enrichment of Pseudomonadota (formerly Proteobacteria) and taxa that include potential opportunistic pathogens such as *Citrobacter* and *Acinetobacter*. BugBase phenotype prediction indicated higher abundance of “Potentially pathogenic” phenotypes in the pond group. These findings suggest that the two aquaculture systems are associated with distinct, coordinated intestinal adaptation patterns: a “stress-resistance-oriented” phenotype in rice paddies characterized by enhanced antioxidant defense, efficient carbohydrate utilization, lower physiological burden, and a more diverse lumen-associated microbiota with greater abundance of potentially beneficial taxa, versus a “growth-oriented” phenotype in ponds characterized by elevated metabolic load, oxidative stress, altered immune status, and opportunistic pathogen enrichment. This study provides a basis for further hypothesis-driven research and may help inform sustainable aquaculture practices, balancing productivity with animal health and welfare.

## 1 Introduction

The red swamp crayfish (*Procambarus clarkii*) is a globally significant farmed freshwater crustacean, supporting a vast aquaculture industry due to its fast growth rate, high adaptability, and substantial economic value (Chen et al., 2024). Native to northeastern Mexico and the south-central United States, this species has been introduced to all continents except Antarctica and is now considered one of the most widely introduced and ecologically impactful invasive freshwater crayfish worldwide, as reflected by its establishment of self-sustaining populations in many non-native regions and its inclusion among the world’s worst invasive alien species (Gherardi, 2006; Lowe et al., 2000). In pursuit of higher productivity, traditional intensive pond monoculture has been widely adopted, characterized by high stocking densities, controlled environments, and formulated feed inputs aimed at maximizing biomass yield (Naylor et al., 2021). Concurrently, driven by principles of sustainability and ecological integration, rice-crayfish co-culture systems have gained substantial traction, particularly in Asia (Ahmed and Garnett, 2011). In such integrated systems, crayfish utilize natural food webs within rice paddies (e.g., plankton, detritus, and insects), reducing reliance on external feed while offering ecosystem services such as pest control and nutrient recycling (Li et al., 2024; Zhang et al., 2023). The choice between these divergent production systems—one prioritizing maximum biomass output through high-input, high-density monoculture, and the other prioritizing system resilience through integrated resource cycling, natural food webs, and reduced external inputs—represents a fundamental strategic decision in modern aquaculture, with direct implications for resource use, environmental impact, and animal welfare (Koretskyi and Kononenko, 2024).

Previous research has begun to delineate some gross differences between intensive and integrated culture systems. Studies typically report that pond-cultured crustaceans achieve superior individual growth rates and final harvest weights, a direct benefit of optimized, nutrient-dense feeding regimes (Ding et al., 2025b; Naylor et al., 2021). In contrast, organisms from integrated or polyculture systems often exhibit different growth patterns and may demonstrate enhanced survival under disease challenge, suggesting a trade-off between maximum growth and robustness (Dong et al., 2024). For instance, research on other aquatic species, such as fish and crustaceans, in integrated systems has highlighted improved water quality parameters and reduced disease incidence (Turcios and Papenbrock, 2014). Furthermore, environmental complexity, such as that found in vegetated rice fields, has been shown to influence behavior and reduce aggression in cultured decapods, potentially lowering stress (Baird et al., 2006). Life-history theory predicts that organisms face trade-offs in allocating limited resources between traits promoting growth, reproduction, maintenance, and survival (Stearns, 1992). An environment favoring rapid biomass gain (like intensive ponds) may select for a “growth-oriented” phenotype, potentially at the expense of investment in stress resilience mechanisms (Shi et al., 2024). Conversely, a more variable, resource-limited environment (like rice paddies) may favor a “resilience-oriented” phenotype with enhanced capabilities for environmental coping (Zhu et al., 2024). However, current evidence remains largely compartmentalized, often focusing on isolated metrics of production or specific stress responses without a unified framework that integrates physiological and microbial dimensions.

The intestine is a critical organ that serves as the primary site for nutrient digestion, absorption, and energy metabolism, while also functioning as a key physical and immunological barrier against pathogens and environmental stressors (Vogt, 2019). In decapod crustaceans, intestinal health is not only determined by the host’s own digestive and immune enzymes but is also shaped by the resident gut microbial community; however, the present study sampled intestinal contents and therefore primarily characterizes lumen-associated rather than resident/symbiotic microbiota. The gut microbiota plays an essential role in host nutrition by facilitating the breakdown of complex carbohydrates, synthesizing essential vitamins, and modulating the host’s immune response (Zhang et al., 2024). A balanced and diverse gut microbiome is increasingly recognized as a hallmark of animal health and resilience, whereas dysbiosis is often linked to increased disease susceptibility and metabolic disorders (Guo et al., 2025). Environmental factors, particularly diet and habitat complexity, are known to exert strong selective pressures on the composition and function of the gut microbial community. For example, dietary shifts from formulated pellets to natural food sources can drastically alter the relative abundance of key bacterial phyla such as *Pseudomonadota*, *Firmicutes*, and *Bacteroidota* (Li et al., 2025). Therefore, a comprehensive evaluation of the intestinal physiological status and its lumen-associated microbiota is essential for understanding how crayfish holistically adapt to different farming environments.

In our previous study on the same batch of experimental animals, we reported that the distinct conditions of intensive pond monoculture and integrated rice-crayfish co-culture are associated with the development of two coherent, organism-wide phenotypic-physiological syndromes in *P. clarkii* (Gao et al., 2026). Specifically, we found that pond-cultured crayfish exhibited a “growth-oriented syndrome” characterized by compact morphology and lipid-anabolic metabolism, but with elevated systemic stress and oxidative damage. In contrast, rice paddy-cultured crayfish displayed a “stress-resistance-oriented syndrome” featuring enhanced antioxidant and immune capacities in the hepatopancreas, underpinned by transcriptomic reprogramming of pathways related to lipid metabolism, detoxification, and endocrine regulation (e.g., upregulation of *SULT1E1*) (Gao et al., 2026). However, while these findings established a systemic framework for understanding environmental adaptation at the organismal and hepatic levels, the corresponding responses of the intestinal tissue—a primary interface between the host, its diet, and the external environment—remain unexplored.

Therefore, the present study was designed to investigate the multi-level adaptive responses of the intestinal system in *P. clarkii* reared under the same two contrasting aquaculture systems. We hypothesized that the distinct nutritional and environmental conditions of the pond and rice paddy systems would not only be associated with divergent systemic physiological states but also with coordinated, system-specific adaptations in intestinal physiology and gut content-associated microbial ecology. Specifically, we predicted that (1) the carbohydrate-rich natural diet in the rice paddy system would enhance intestinal carbohydrate digestion (amylase activity) and bolster antioxidant defenses, whereas the high-protein/high-lipid formulated feed in the pond system would elevate lipase and protease activities; (2) the rice paddy system would be associated with enhanced intestinal mucosal immune barrier function, as indicated by higher AKP and ACP activities, whereas the pond system would be associated with higher Na^+^/K^+^-ATPase activity, reflecting a greater intestinal absorptive workload and energy demand; and (3) the gut content-associated microbial community structure and predicted metabolic functions would differ markedly between the two systems, with the rice paddy environment being associated with higher diversity and a microbiome enriched in potentially beneficial, carbohydrate-metabolizing taxa, while the pond system would show enrichment of opportunistic or potentially pathogenic bacteria.

To test these hypotheses, we conducted a comprehensive comparative analysis integrating intestinal enzymatic activity assays (digestive, antioxidant, immune barrier, and energy metabolism enzymes) with high-throughput 16S rRNA gene gut content-associated microbiota. By synthesizing these physiological and microbial data, our study aims to provide a mechanistic understanding of how the intestine contributes to the overall environmental adaptation of the red swamp crayfish. This work will complement our previous hepatopancreatic transcriptomic findings (Gao et al., 2026) and offer a more complete, multi-organ perspective on the biological trade-offs inherent to different aquaculture production models. The results are expected to provide scientific insights for optimizing sustainable farming practices, balancing productivity with animal health and welfare.

## 2 Materials and methods

### 2.1 Animal rearing and sample collection

This study was conducted using the same batch of juvenile red swamp crayfish (*Procambarus clarkii*) and the identical experimental design as described in our previous study on hepatopancreatic transcriptomics (Gao et al., 2026). Briefly, uniform juvenile crayfish (initial body weight 5.0±0.5g ) were randomly assigned to two distinct culture systems for a 60-day trial: a traditional intensive pond monoculture system (Pond group, C) and an integrated rice-crayfish co-culture system (Rice paddy group, T). Each group consisted of three independent replicate units, with a standardized stocking density of 15 individuals per m^2^. The pond group was fed a commercial formulated feed twice daily to satiation (approximately 3% of biomass per day), while the rice paddy group primarily relied on natural food webs within the rice field ecosystem and received a daily supplement of only 30% of the pond feed amount (equivalent to approximately 0.9% of biomass). Water quality parameters were monitored weekly and maintained within optimal ranges for crayfish culture (Gao et al., 2026).

At the end of the 60-day culture period, crayfish were harvested. For intestinal enzyme activity assays, 9 individuals per replicate unit were randomly selected (*n* = 27 per group). For 16S rRNA gene sequencing, 3 individuals per replicate unit were randomly selected, yielding 9 individuals per group (total *n* = 18). All selected individuals were anesthetized on ice for 10–15 min and then euthanized by rapid freezing at −80 °C before dissection. The entire intestinal tract, from the gastric mill to the anus, was carefully removed. The intestinal lumen contents were gently flushed with sterile phosphate-buffered saline (PBS, 0.1M, pH 7.4), collected into sterile centrifuge tubes, and immediately flash-frozen in liquid nitrogen before storage at −80 °C for subsequent 16S rRNA gene sequencing. Following removal of the contents, the intestinal tissue itself was cleaned, snap-frozen in liquid nitrogen, and stored at −80 °C for subsequent enzymatic activity assays. Because the crayfish intestine is small and tissue-attached microbial biomass is often limited, intestinal contents were used for 16S rRNA sequencing to obtain sufficient microbial DNA. This approach characterizes the lumen-associated microbial community, which may include both resident and transient taxa derived from diet, water, and sediment. Therefore, the sequencing results are interpreted as gut content-associated microbiota rather than as true symbiotic or tissue-attached microbiota.

### 2.2 Intestinal enzyme activity assays

Frozen intestinal tissue samples were homogenized in ice-cold PBS (0.1M, pH 7.4) using a tissue homogenizer. The homogenates were then centrifuged at 10,000×g for 15 min at 4 °C, and the resulting supernatants were collected for enzymatic analyses. Total protein concentration in the supernatants was determined using a Bradford protein assay kit (Beyotime Biotechnology, Shanghai, China) to normalize enzyme activities.

The activities of key digestive enzymes were measured using commercially available assay kits: amylase (Amylase Assay Kit, catalog no. C016-1-1, starch-iodine colorimetric method), lipase (Lipase Assay Kit, catalog no. A054-2-1, colorimetric method), and total protease (Protease Assay Kit, catalog no. A080-2-2, UV colorimetric method) (Nanjing Jiancheng Bioengineering Institute, China). For antioxidant capacity, superoxide dismutase (SOD, catalog no. A001-3-2, WST-1 method), catalase (CAT, catalog no. A007-2-1, visible light method), and glutathione peroxidase (GSH-Px, catalog no. A005-1-2) activities, as well as malondialdehyde (MDA, catalog no. A003-1-2, TBA method) content, were determined using corresponding colorimetric assay kits (Nanjing Jiancheng Bioengineering Institute, China). Additionally, intestinal mucosal immune barrier-related enzymes, alkaline phosphatase (AKP, catalog no. A059-1-1, visible light colorimetric method) and acid phosphatase (ACP, catalog no. A060-1-1, visible light colorimetric method), were measured using commercially available kits (Nanjing Jiancheng Bioengineering Institute, China). The activity of Na^+^/K^+^-ATPase (catalog no. A070-2-1, ultra-micro Na^+^K^+^-ATPase assay kit) was determined using a specific assay kit (Nanjing Jiancheng Bioengineering Institute, China).

### 2.3 DNA extraction, 16S rRNA gene amplification, and sequencing

Microbial genomic DNA was extracted from the intestinal content samples (*n* = 9 per group, total *n* = 18) using the E.Z.N.A.® Soil DNA Kit (Omega Bio-tek, Norcross, GA, USA) according to the manufacturer’s protocol. The quality and concentration of the extracted DNA were assessed via 1% agarose gel electrophoresis and a NanoDrop 2000 spectrophotometer (Thermo Fisher Scientific, Waltham, MA, USA). The V3-V4 hypervariable regions of the bacterial 16S rRNA gene were amplified using the universal primers 338F (5′-ACTCCTACGGGAGGCAGCAG-3′) (Lane, 1991) and 806R (5′-GGACTACHVGGGTWTCTAAT-3′) (Caporaso et al., 2011; Walters et al., 2016). The PCR reactions were performed in a total volume of 25 μL, containing 12.5 μL of 2 × Phusion High-Fidelity PCR Master Mix (New England Biolabs, Ipswich, MA, USA), 0.5 μL of each primer (10 μM), and 10 ng of template DNA. The thermal cycling program was as follows: initial denaturation at 98 °C for 30 s; followed by 30 cycles of denaturation at 98 °C for 10 s, annealing at 54 °C for 30 s, and extension at 72 °C for 45 s; with a final extension at 72 °C for 10 min. The resulting PCR products were purified using an Agencourt AMPure XP kit (Beckman Coulter, Brea, CA, USA) and quantified using a Quant-iT PicoGreen dsDNA Assay Kit (Invitrogen, Carlsbad, CA, USA). Equimolar amounts of the purified amplicons from each sample were pooled and paired-end sequenced (2 × 300 bp) on the Illumina MiSeq platform (Illumina, San Diego, CA, USA) by Majorbio Bio-Pharm Technology Co., Ltd. (Shanghai, China).

### 2.4 Bioinformatics and statistical analysis of gut content-associated microbiota

The raw sequencing reads were demultiplexed and quality-filtered using QIIME 2 (version 2023.9) (Bolyen et al., 2019) with the DADA2 plugin (Callahan et al., 2016). The parameters for quality filtering included truncation of reads at positions where the average quality score dropped below 20, with a sliding window of 10 bp. After chimera removal, the resulting high-quality amplicon sequence variants (ASVs) were used for subsequent analyses. Taxonomic assignment of ASVs was performed against the SILVA database (version 138.2) (Quast et al., 2012) using a pre-trained Naive Bayes classifier with a confidence threshold of 0.7.

For alpha diversity analysis, indices including Shannon, Simpson, Chao1, ACE, and Coverage were calculated based on the ASV-level abundance data. Differences in alpha diversity indices between the pond (C) and rice paddy (T) groups were assessed using independent samples *t*-tests in SPSS (version 26.0). For beta diversity analysis, principal coordinate analysis (PCoA) was performed based on Bray-Curtis distances. The statistical significance of the differences in microbial community structure between the two groups was evaluated using Permutational Multivariate Analysis of Variance (PERMANOVA) and Analysis of Similarities (ANOSIM) with 999 permutations. The relative abundance of microbial communities at the phylum and genus levels was calculated and visualized for each group. Linear Discriminant Analysis Effect Size (LEfSe) analysis (LDA score > 3, *p* < 0.05) (Segata et al., 2011) and Metastats analysis (White et al., 2009) were used to identify differentially abundant taxa between the two groups.

To explore the potential functional and phenotypic properties of the gut content-associated microbial communities, PICRUSt2 (Douglas et al., 2020) and BugBase (Ward et al., 2017) were applied to the ASV sequences. Predicted metagenomic functions were mapped to the Kyoto Encyclopedia of Genes and Genomes (KEGG) database (Kanehisa et al., 2016), and differences in the relative abundances of KEGG pathways were analyzed using STAMP software (Parks et al., 2014). These analyses were exploratory and predictive, and their results were not used as the primary basis for functional or health claims because the dataset is based on gut contents and water/sediment microbiota were not characterized.

### 2.5 Statistical analysis

All statistical analyses for the intestinal enzyme activity data were performed using SPSS software (version 26.0, IBM Corp, Armonk, NY, USA). Data are presented as mean ± standard error (SE). Independent samples *t*-tests were used to compare the differences in physiological and enzymatic indices between the pond (C) and rice paddy (T) groups. A significance threshold of *p* < 0.05 was applied unless otherwise indicated. Bioinformatic and statistical analyses for the 16S rRNA sequencing data were conducted using the online Majorbio Cloud Platform^1^ and the R software (version 4.0.5). Detailed data on sequencing quality and results are provided in the Supplementary Figures S1–S8 and Supplementary Tables S1–S3.

## 3 Results

### 3.1 Intergroup differences in intestinal digestive enzyme activities

The activities of intestinal digestive enzymes (amylase, lipase, and total protease) were compared between the pond-cultured (C) and rice paddy-cultured (T) groups (Figures 1A–C). The rice paddy group exhibited significantly higher amylase activity compared to the pond group (*p* < 0.001; Figure 1C), suggesting enhanced carbohydrate digestion capacity in crayfish from the integrated rice-crayfish system. This elevated amylase activity is consistent with the carbohydrate-rich composition of the natural food resources (approximately 47.3% carbohydrates) available in the rice paddy ecosystem (Gao et al., 2026). In contrast, the pond group showed significantly higher lipase and total protease activities than the rice paddy group (*p* < 0.001 and *p* < 0.01, respectively; Figures 1A,B), corresponding to the high-fat (6.8%) and high-protein (32.5%) formulated feed provided in the intensive pond system (Gao et al., 2026).

**Figure 1:**
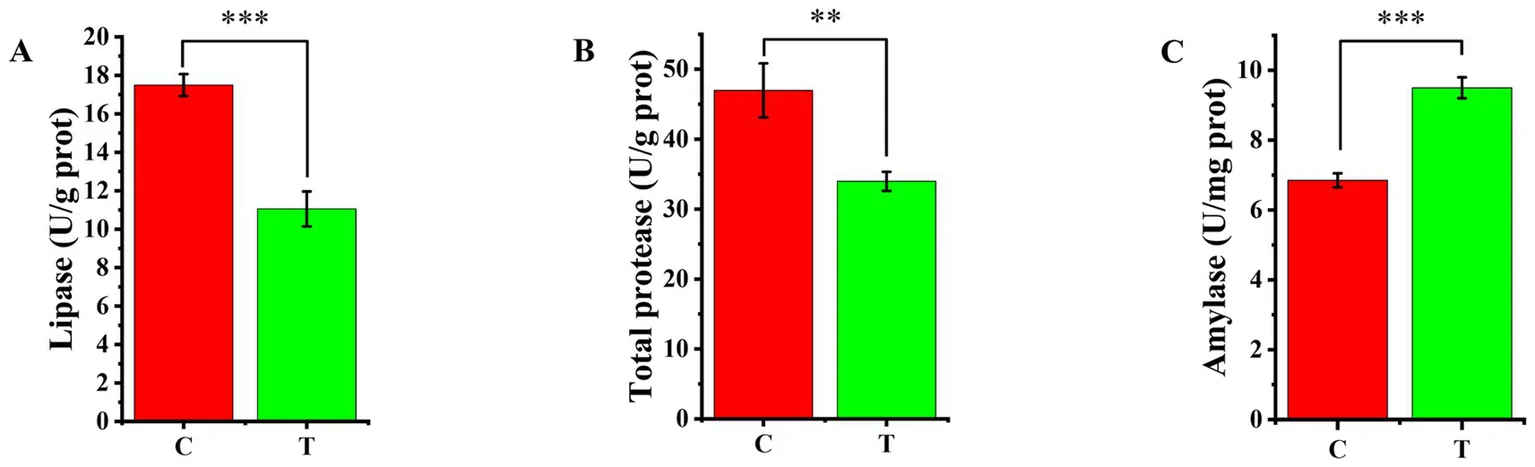
Intestinal digestive enzyme activities of *Procambarus clarkii* reared in pond (C) and rice paddy (T) systems **(A)** Lipase activity (U/g prot); **(B)** Total protease activity (U/g prot); **(C)** Amylase activity (U/mg prot). Bars represent mean ± SE (*n* = 9 per group). **p < 0.01, ***p < 0.001 (independent samples *t*-test).

### 3.2 Intergroup differences in intestinal antioxidant enzyme activities and oxidative damage

Antioxidant enzyme activities (SOD, CAT, GSH-Px) and oxidative damage marker (MDA) were compared between the pond-cultured (C) and rice paddy-cultured (T) groups (Figures 2A–D). The rice paddy group exhibited significantly higher activities of SOD, CAT, and GSH-Px compared to the pond group (*p* < 0.001 for all; Figures 2A–C), suggesting enhanced antioxidant defense capacity in crayfish from the integrated rice-crayfish system. In contrast, the MDA content, a biomarker of lipid peroxidation, was significantly lower in the rice paddy group than in the pond group (*p* < 0.001; Figure 2D), suggesting reduced oxidative damage under the rice paddy farming conditions. Notably, the same trends were also observed in the hepatopancreas of these crayfish in our previous study (Gao et al., 2026), suggesting a systemic enhancement of antioxidant capacity in the rice paddy system.

**Figure 2:**
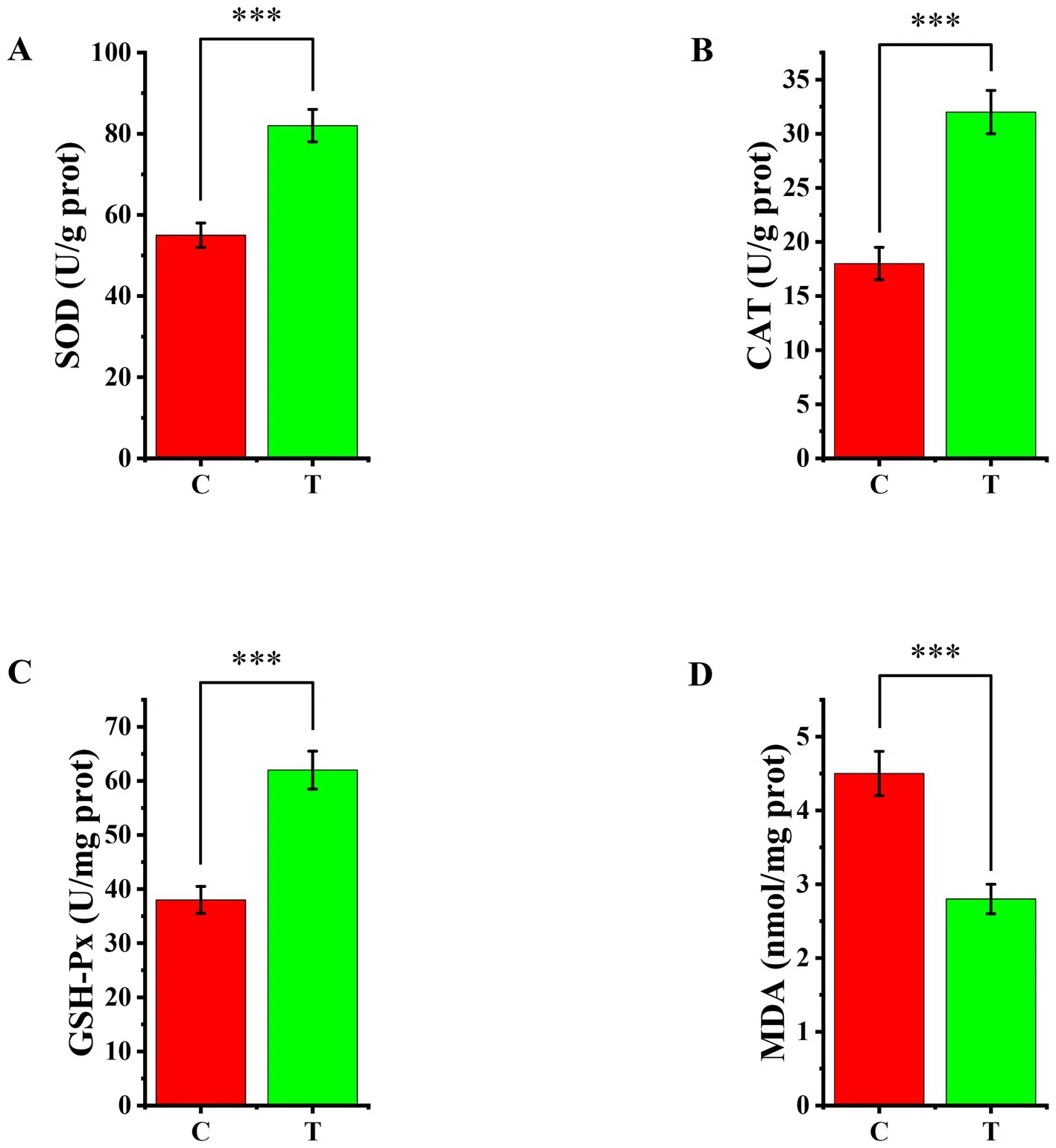
Intestinal antioxidant enzyme activities and oxidative damage marker of *Procambarus clarkii* reared in pond (C) and rice paddy (T) systems. **(A)** Superoxide dismutase (SOD) activity (U/g prot); **(B)** Catalase (CAT) activity (U/g prot); **(C)** Glutathione peroxidase (GSH-Px) activity (U/mg prot); **(D)** Malondialdehyde (MDA) content (nmol/mg prot). Bars represent mean ± SE (*n* = 9 per group). *** Indicates *p* < 0.001 (independent samples *t-t*est).

### 3.3 Intergroup differences in intestinal immune barrier enzyme activities

Intestinal non-specific immune barrier enzyme activities (AKP and ACP) were compared between the pond-cultured (C) and rice paddy-cultured (T) groups (Figures 3A,B). The rice paddy group exhibited significantly higher activities of both AKP and ACP compared to the pond group (*p* < 0.001 for both; Figures 3A,B).

**Figure 3:**
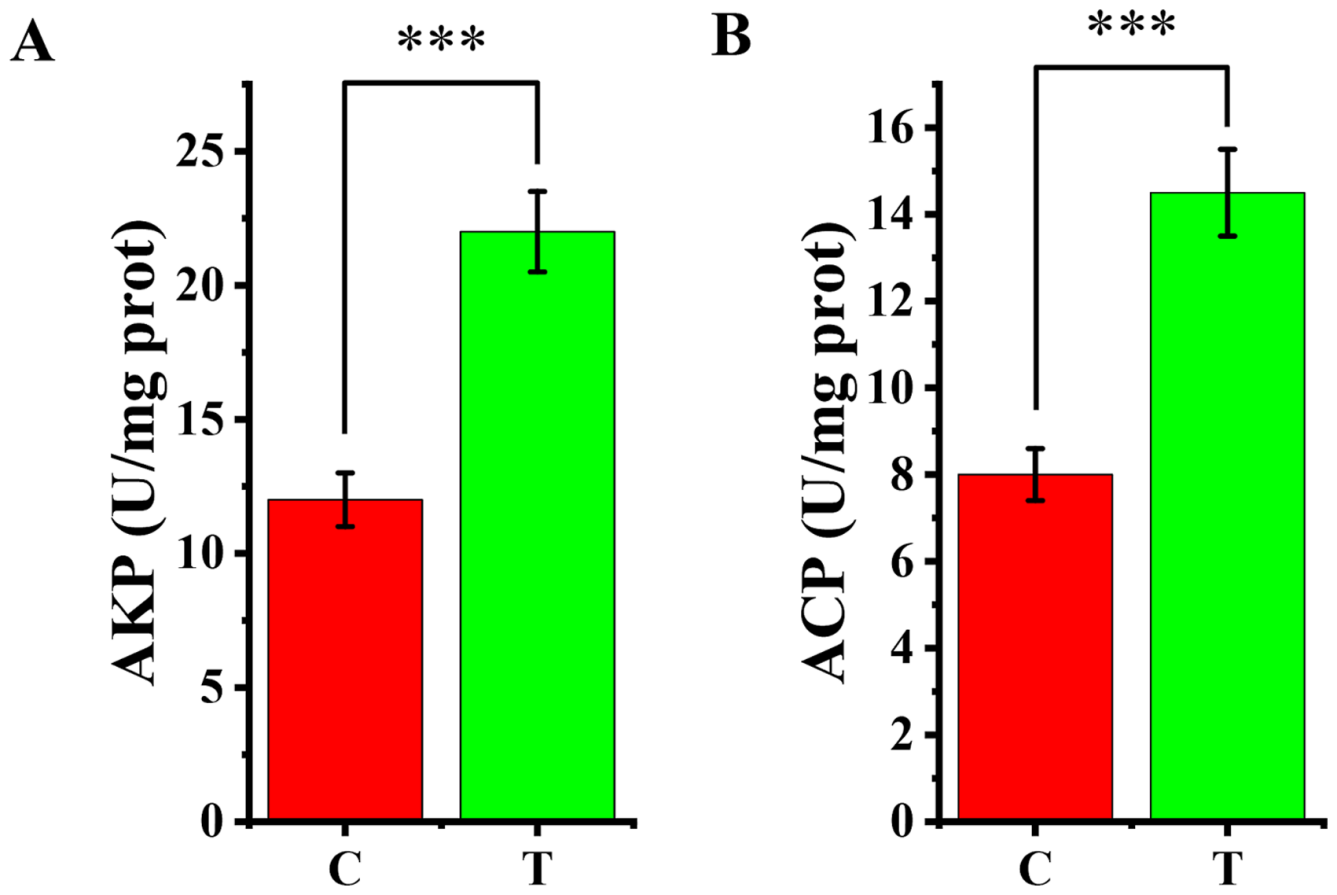
Intestinal immune barrier enzyme activities of *Procambarus clarkii* reared in pond (C) and rice paddy (T) systems. **(A)** Alkaline phosphatase (AKP) activity (U/mg prot); **(B)** Acid phosphatase (ACP) activity (U/mg prot). Bars represent mean ± SE (*n* = 9 per group). *** Indicates *p* < 0.001 (independent samples *t*-test).

### 3.4 Intergroup differences in intestinal energy metabolism enzyme activity

Intestinal Na^+^/K^+^-ATPase activity, a key enzyme reflecting epithelial energy metabolism and active ion transport, was compared between the pond-cultured (C) and rice paddy-cultured (T) groups (Figure 4). The pond group exhibited significantly higher Na^+^/K^+^-ATPase activity compared to the rice paddy group (*p* < 0.001), suggesting enhanced intestinal absorptive function and energy demand under the intensive pond culture conditions. In contrast, the lower Na^+^/K^+^-ATPase activity observed in the rice paddy group suggests a relatively reduced intestinal transport workload, consistent with the lower nutrient availability in the natural food-based rice paddy system.

**Figure 4:**
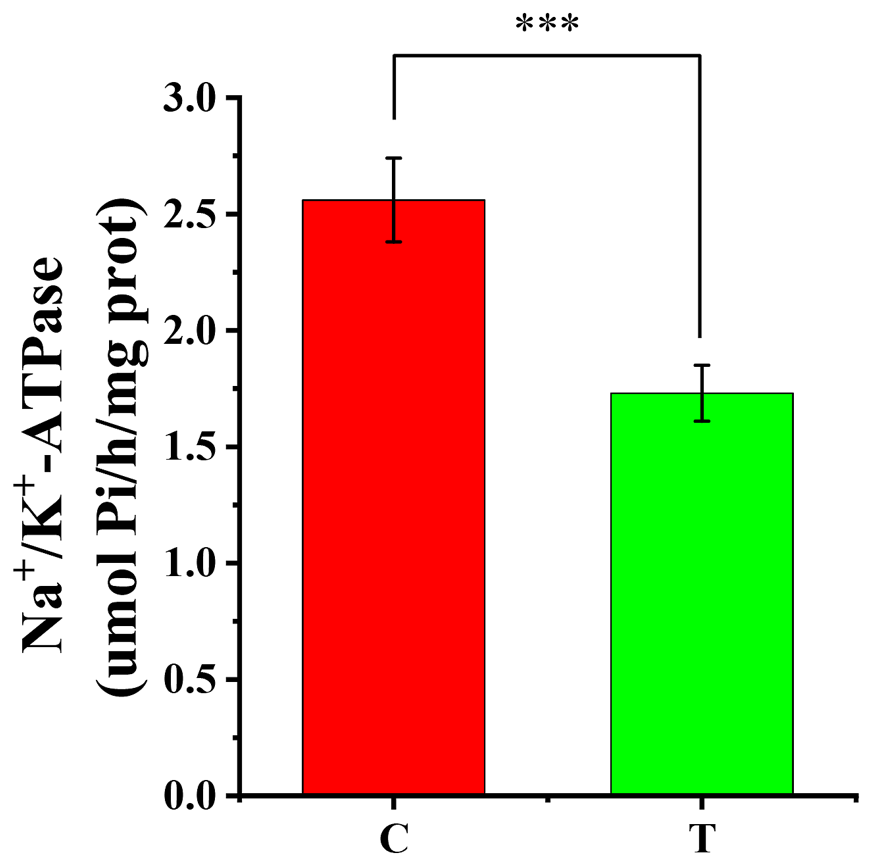
Comparison of intestinal Na^+^/K^+^-ATPase activity (μmol P_i_/h/mg prot) between the pond-cultured (C) and rice paddy-cultured (T) groups. Bars represent mean ± SE (*n* = 9 per group). *** Indicates significant difference at *p* < 0.001 (independent samples *t-t*est).

### 3.5 Intergroup differences in gut content-associated microbial community composition and diversity

#### 3.5.1 Sequencing data quality and alpha diversity

A total of 1,439,437 raw reads were generated from the 18 intestinal content samples (*n* = 9 per group) via 16S rRNA sequencing. After quality control and denoising, 1,207,531 clean reads were obtained, with an average of 67,085 reads per sample (ranging from 65,687 to 68,974; Supplementary Table S1). Following chimera removal, 1,091,470 high-quality non-chimeric sequences were retained for downstream analysis, averaging 60,637 sequences per sample (ranging from 55,838 to 65,698; Supplementary Table S1). Both the rarefaction curves and the Shannon index curves reached plateau phases (Supplementary Figures S1, S2), indicating that the sequencing depth adequately covered the microbial diversity of the samples and was sufficient for subsequent analyses.

A total of 3,321 amplicon sequence variants (ASVs) were obtained following DADA2 denoising, with ASV numbers per sample ranging from 175 to 463 (Supplementary Table S2). The Venn diagram (Figure 5) revealed that the rice paddy group (T) and the pond group (C) shared 387 ASVs, while 1,668 ASVs were unique to the T group and 1,266 ASVs were unique to the C group, suggesting a certain degree of difference in gut microbial community composition between the two groups.

**Figure 5:**
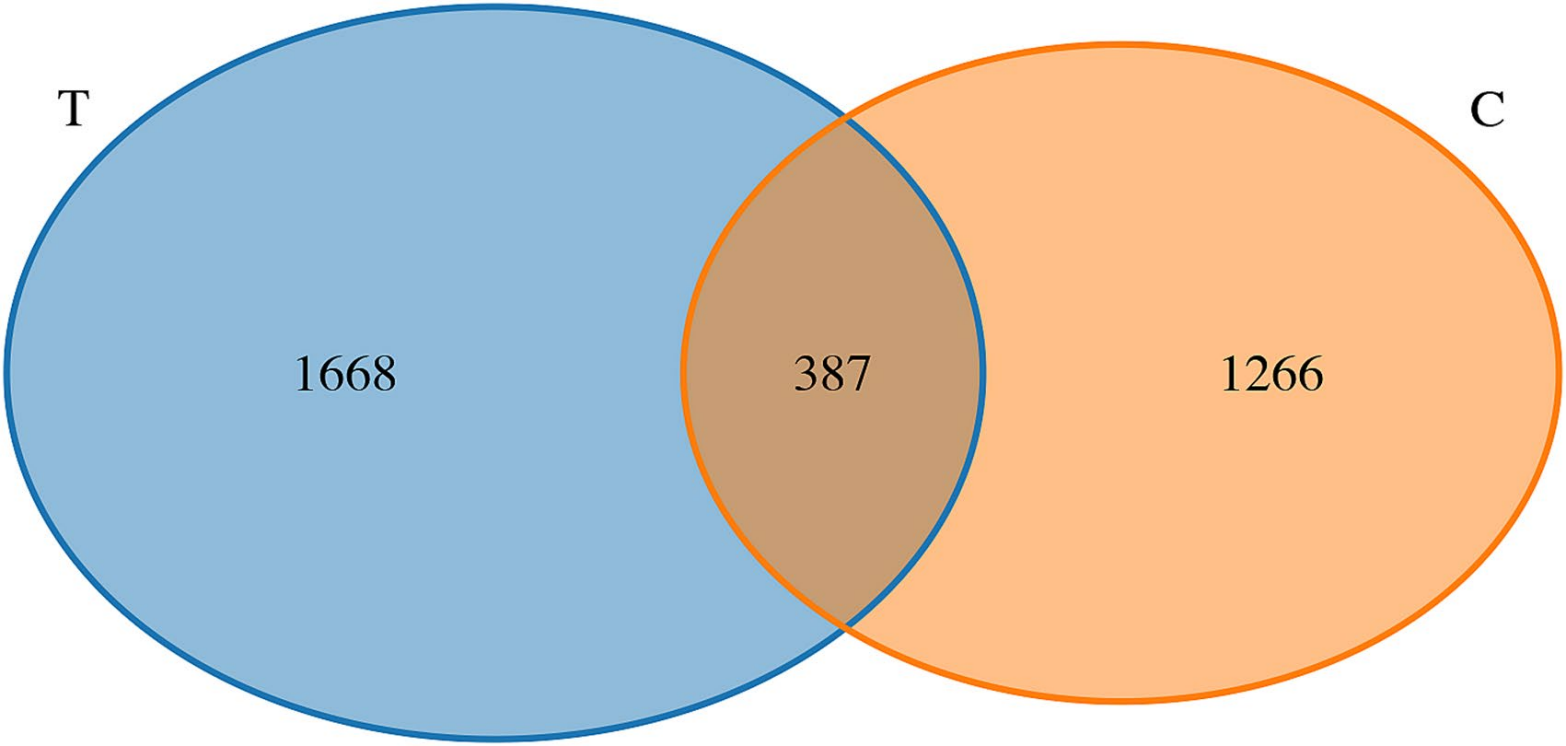
Venn diagram illustrating shared and unique ASVs between the rice paddy-cultured (T) and pond-cultured (C) groups. Different colors represent different groups; overlapping regions indicate shared ASVs, and non-overlapping regions indicate group-specific ASVs.

Alpha diversity analysis (Table 1) showed that the rice paddy group (T) exhibited higher mean values for the Shannon index (approx. 5.84), Simpson index (approx. 0.952), Chao1 index (approx. 376.2), and ACE index (approx. 379.0) compared to the pond group (C) (Shannon approx. 5.08, Simpson approx. 0.929, Chao1 approx. 311.6, ACE approx. 316.1). Significant differences between the two groups were observed for the Shannon and Chao1 indices (*t*-test, *p* < 0.05), while the Simpson and ACE indices also showed a trend of being higher in the rice paddy group.

**Table 1:** Permutational multivariate analysis of variance (PERMANOVA) results comparing gut microbial community structure between the pond-cultured (C) and rice paddy-cultured (T) groups based on Bray–Curtis distances.

diffs	Df	SumsOfSqs	MeanSqs	F. Model	*R* ^2^	Pvalue	Psignf
T VS C	1	0.488031893491	0.488031893491	1.39078949083	0.0799727632585	0.001	**

diffs, comparison groups; Df, degrees of freedom; SumsOfSqs, sum of squares; MeanSqs, mean squares; F.Model, F-statistic; R^2^, proportion of variance explained by the grouping factor; Pvalue, significance level; Psignf, significance designation (**p < 0.01).

The Coverage index exceeded 0.9998 for all samples, confirming that the sequencing results accurately reflect the microbial community composition of each sample.

#### 3.5.2 Beta diversity analysis

Principal coordinate analysis (PCoA) based on Bray–Curtis distances (Figure 6) revealed a clear separation between the rice paddy group (T) and the pond group (C) along the first principal coordinate axis (PC1, explaining 26.83% of the variance), indicating a systematic difference in gut content-associated microbial community structure between the two farming systems.

**Figure 6:**
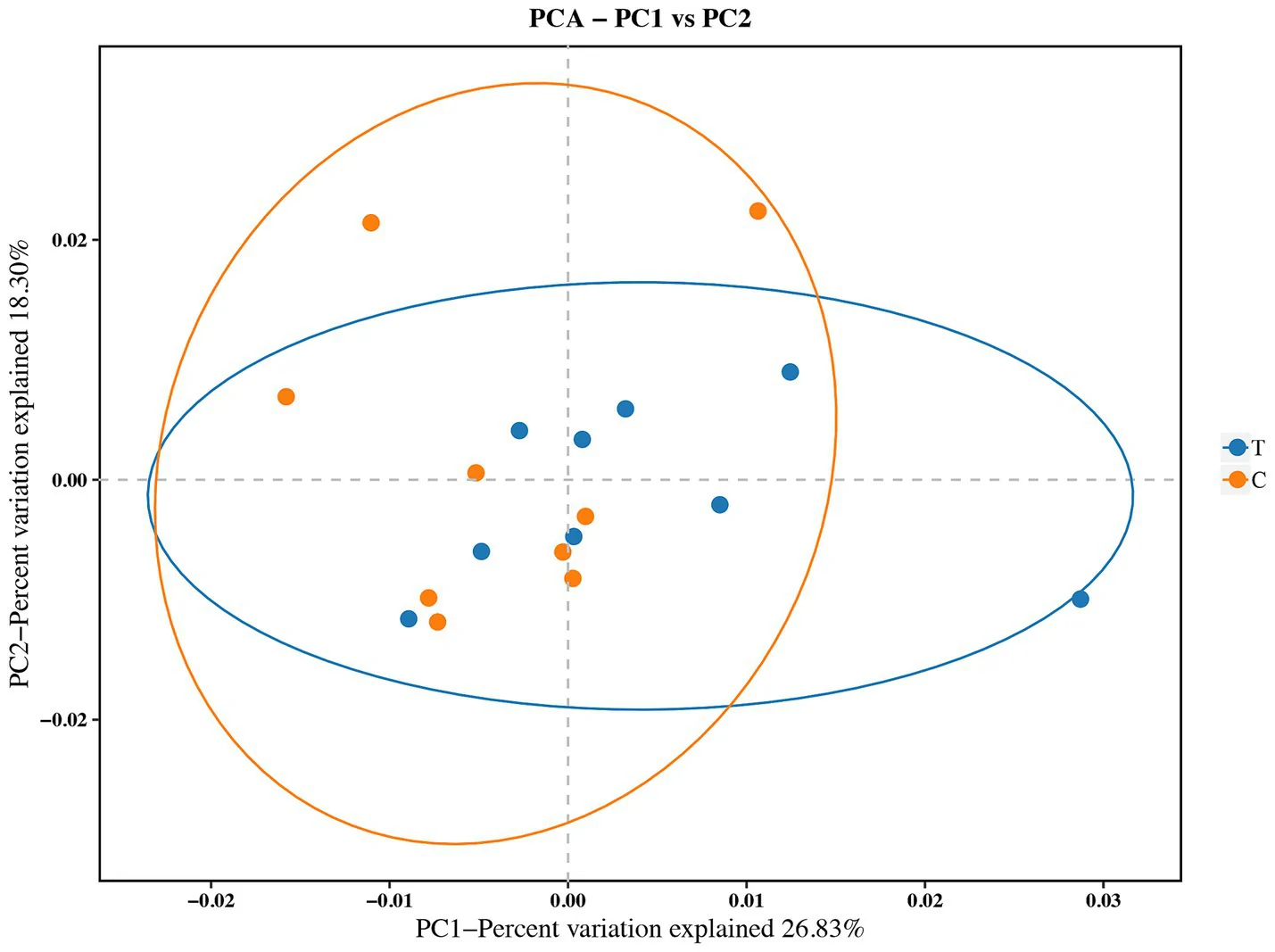
Principal coordinate analysis (PCoA) of gut microbial communities in the rice paddy-cultured (T) and pond-cultured (C) groups. Each point represents an individual sample, with different colors indicating different groups. Ellipses represent 95% confidence intervals around the group centroids. The percentages on the axes indicate the proportion of variance explained by each principal coordinate.

PERMANOVA results showed that the farming mode was significantly associated with gut microbial content-associated microbial community composition, explaining 7.99% of the total variance (*R*^2^ = 0.07997, F.Model = 1.3908, *p* = 0.001). Although the explained variance was relatively low, which is common in gut microbiome studies due to high inter-individual variability, the separation between the two groups was statistically significant. ANOSIM (analysis of similarities) results (Table 2) further supported this conclusion, with an *R*-value of 0.3354 (0.25 < *R* < 0.5, indicating a moderate degree of difference between groups) and a *p*-value of 0.001 (*p* < 0.05).

**Table 2:** Analysis of similarities (ANOSIM) results comparing gut microbial community structure between the pond-cultured (C) and rice paddy-cultured (T) groups based on Bray–Curtis distances.

diffs	*R*	Pvalue	Psignf
T VS C	0.3353909	0.001	*

diffs, comparison groups; R, ANOSIM R-statistic (values > 0 indicate greater dissimilarity between groups than within groups); Pvalue, significance level; Psignf, significance designation (*p < 0.05).

Collectively, these multi-faceted beta diversity analyses consistently showed that the gut content-associated microbial community structure of crayfish differs significantly between the pond and rice paddy groups.

#### 3.5.3 Community composition and differential abundance analysis

At the phylum level (Figure 7), the dominant bacterial taxa in both groups were *Pseudomonadota*, *Firmicutes*, and *Bacteroidota*, which collectively accounted for more than 80% of the total microbial community in both groups. Additionally, lower-abundance phyla including *Patescibacteria*, *Actinobacteriota*, *Cyanobacteria*, *Verrucomicrobiota*, *Spirochaetota*, and *Deinococcota* were detected in both groups. In terms of relative abundance, the rice paddy group (T) exhibited a higher proportion of *Bacteroidota* compared to the pond group (C), whereas the pond group showed a higher relative abundance of *Pseudomonadota*.

**Figure 7:**
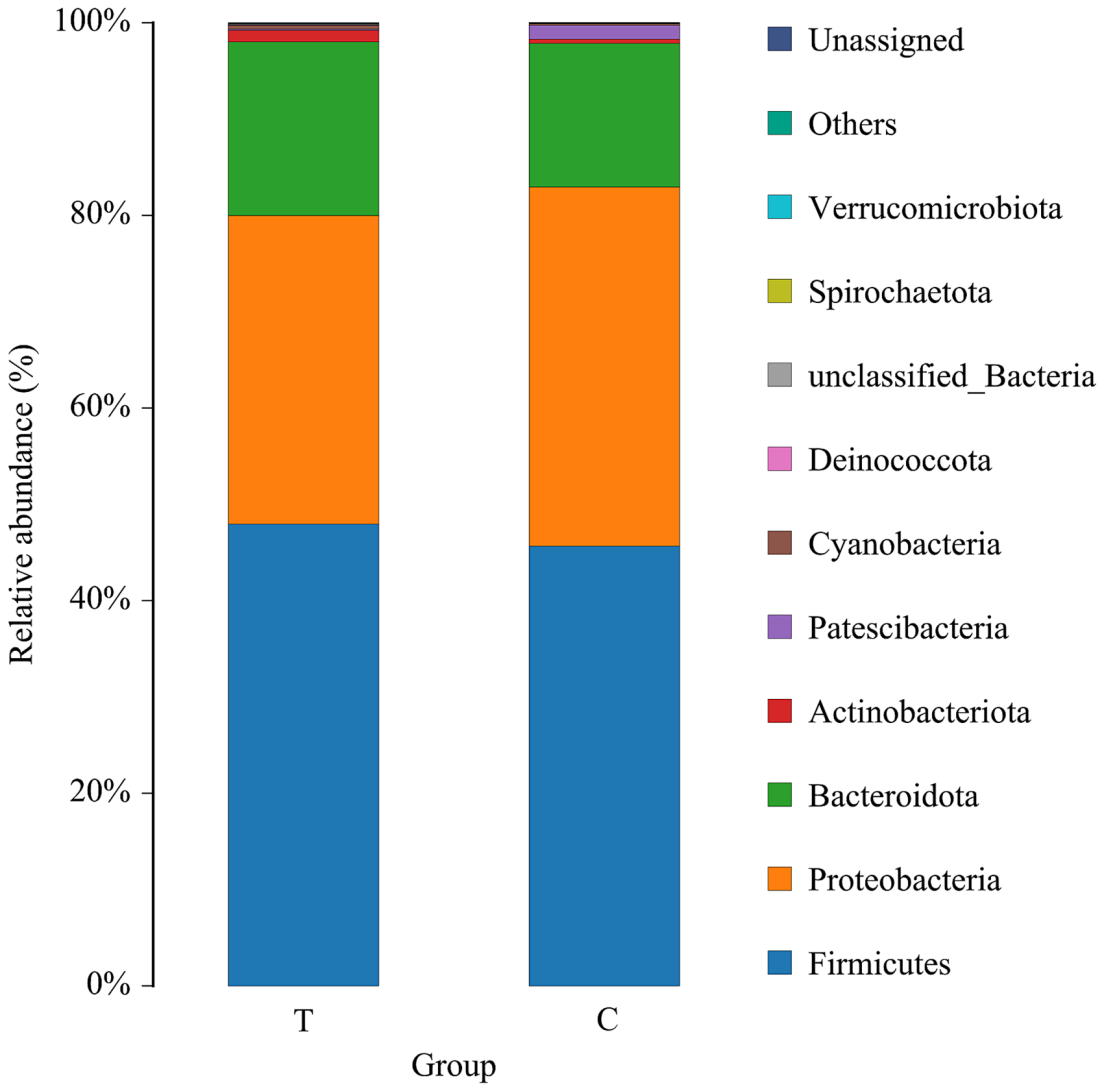
Taxonomic composition of gut microbial communities at the phylum level in the rice paddy-cultured (T) and pond-cultured (C) groups. Each color represents a different phylum, and the height of each bar indicates the relative abundance of each taxon within each group. Taxa with relative abundance below the top 10 across all samples are grouped as “Others,” and sequences that could not be assigned to any known phylum are labeled as “Unassigned”.

At the genus level (Figure 8), Bacteroides and Candidatus_Bacilloplasma were more abundant in the rice paddy group (T), whereas Citrobacter and Acinetobacter, together with other members of the orders Enterobacterales and Pseudomonadales, were enriched in the pond group (C). Genera such as *unclassified_bacterium_PM5_3*, *ZOR0006*, *Shewanella*, and *unclassified_Weeksellaceae* were detected at moderate relative abundances in both groups.

**Figure 8:**
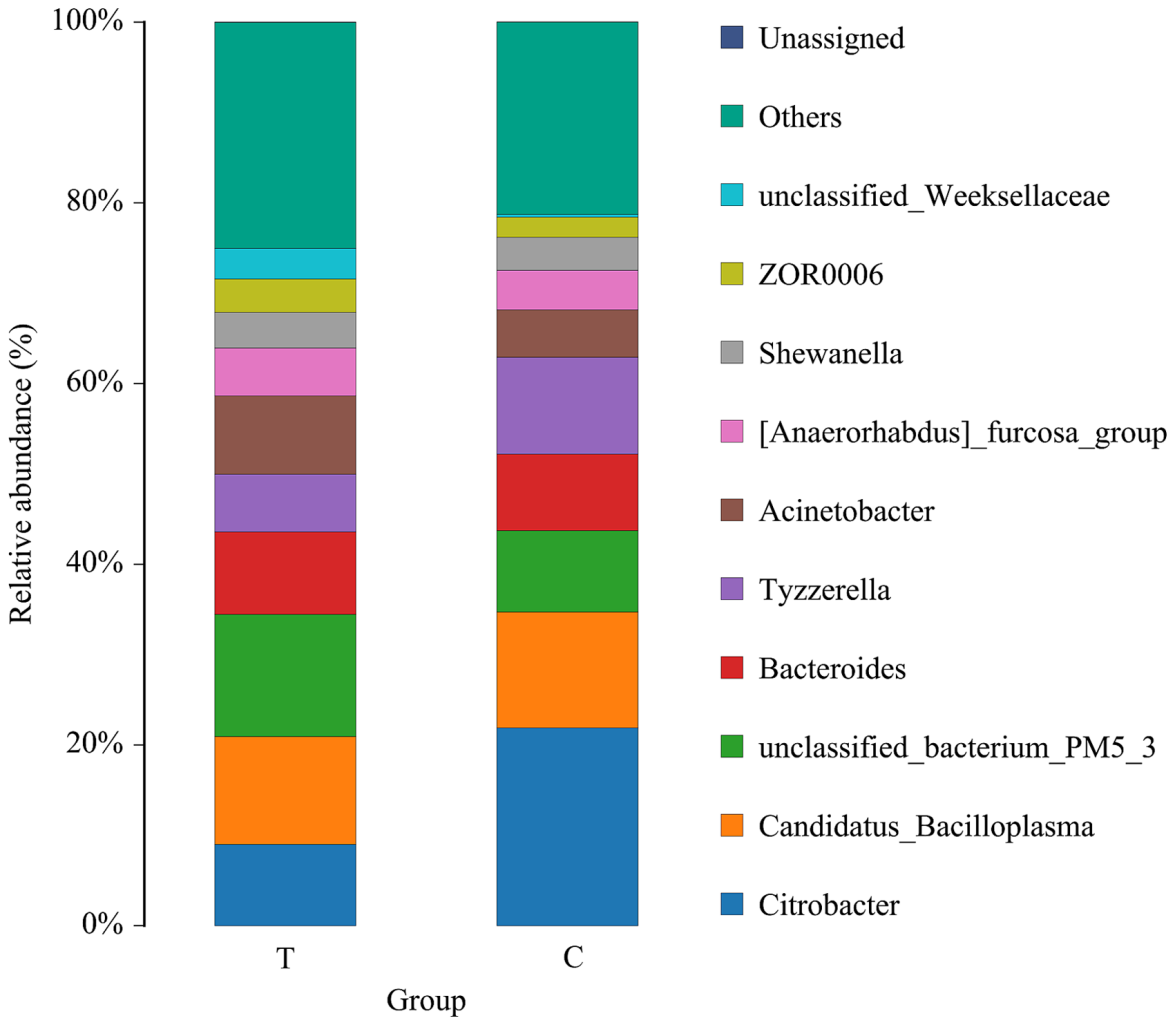
Taxonomic composition of gut microbial communities at the genus level in the rice paddy-cultured (T) and pond-cultured (C) groups. Each color represents a different genus, and the height of each bar indicates the relative abundance of each taxon within each group. Genera with relative abundance below the top 10 across all samples are grouped as “Others,” and sequences that could not be classified to a known genus are labeled as “Unassigned”.

LEfSe analysis (LDA > 3, *p* < 0.05; Figures 9, 10) revealed distinct biomarker taxa associated with each group. The pond group (C) was characterized by enrichment of the orders *Enterobacterales* and *Pseudomonadales*, families *Enterobacteriaceae* and *Moraxellaceae*, genera *Citrobacter* and *Acinetobacter*, and species *Citrobacter braakii*, *Citrobacter freundii*, and *Acinetobacter lwoffii* (Figure 9). The cladogram (Figure 10) further visualized that the pond group biomarkers were primarily concentrated within *Enterobacterales* and *Pseudomonadales* and their affiliated families and genera, whereas the rice paddy group (T) biomarkers were predominantly associated with the phylum *Bacteroidota* and its subordinate taxa. The abundance distribution of *Enterobacteriaceae* between the two groups is shown in Figure 9.

**Figure 9:**
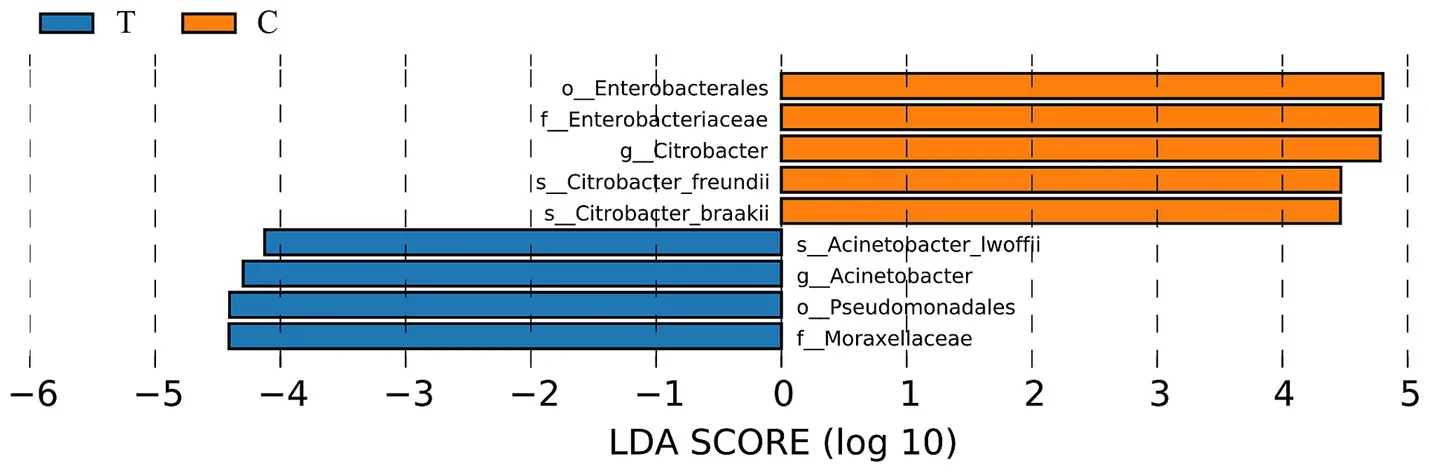
Linear discriminant analysis (LDA) effect size (LEfSe) scores of differentially abundant taxa between the rice paddy-cultured (T) and pond-cultured (C) groups. The histogram displays LDA scores (log_10_) for taxa with significant differential abundance (LDA > 3, *p* < 0.05). Bar length indicates the magnitude of the effect size, and bar color indicates the group with higher relative abundance of the corresponding taxon.

**Figure 10:**
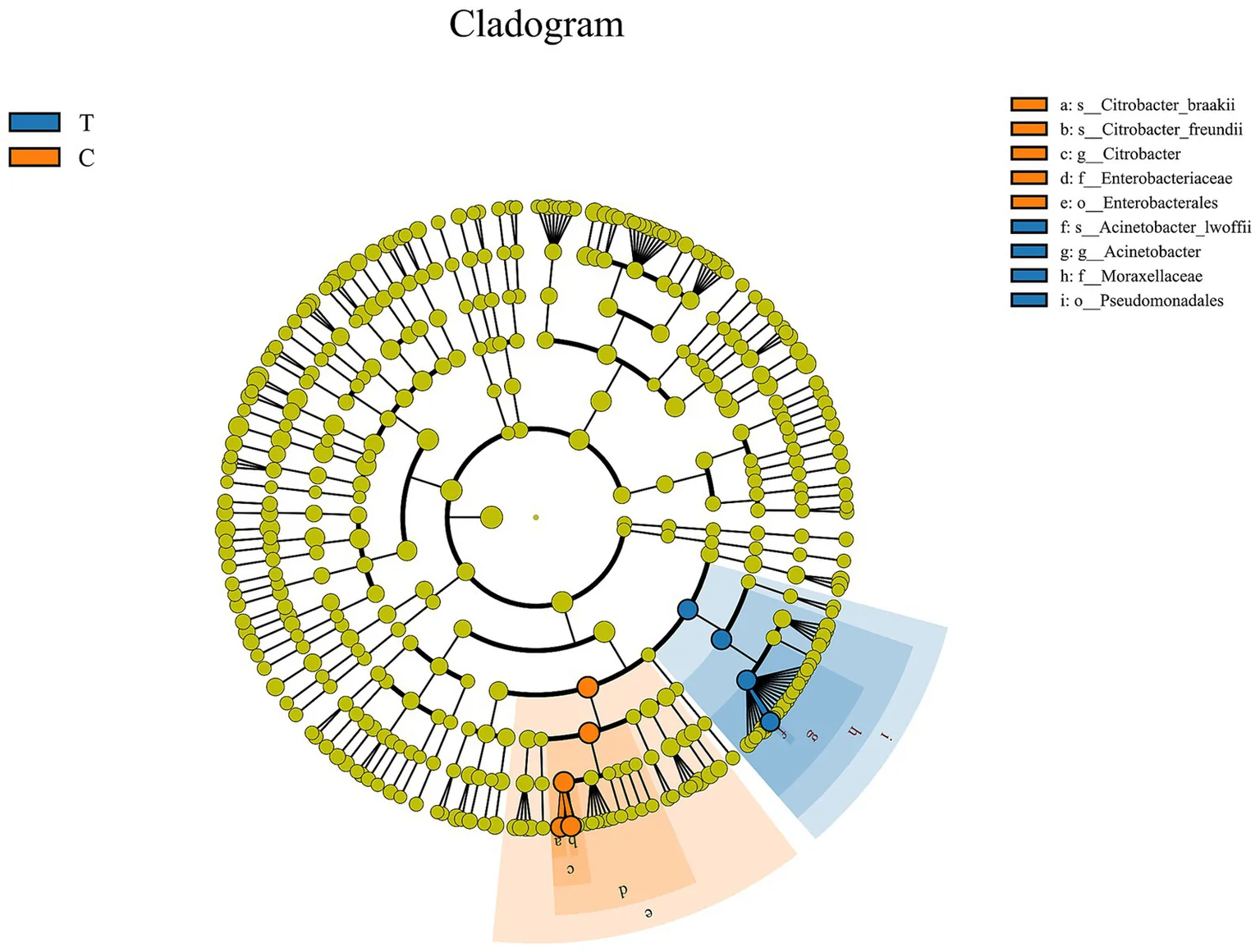
Cladogram generated by LEfSe analysis showing the phylogenetic distribution of differentially abundant taxa between the rice paddy-cultured (T) and pond-cultured (C) groups. Circles radiating from the center to the periphery represent taxonomic levels from phylum to species. Each circle at a given taxonomic level represents a taxon, with circle diameter proportional to its relative abundance. Non-significant taxa are colored yellow, while significant taxa are colored according to the group in which they are most abundant (T or C).

Metastats analysis (Figure 11 and Supplementary Table S3) identified a total of 81 genera with significantly different abundances between the two groups (*p* < 0.05). Among these, *Acidovorax*, *Aquaspirillum*, *Bauldia*, *Crenothrix*, *Dermacoccus*, *Ferruginibacter*, *Halomonas*, *Hydrogenophaga*, *Kineosporia*, *Lacihabitans*, and *Leifsonia* were significantly enriched in the rice paddy group (T) (*p* < 0.001), whereas *Actinoplanes*, *Agromyces*, *Anaerococcus*, *Desulfobulbus*, *Ezakiella*, *Facklamia*, *Finegoldia*, *Micromonospora*, *Plesiomonas*, and *Saccharibacillus* were significantly enriched in the pond group (C) (*p* < 0.001). Notably, most of the T-enriched genera were undetectable (mean abundance = 0) in the pond group, suggesting they may represent low-abundance taxa uniquely associated with the rice-crayfish co-culture system.

**Figure 11:**
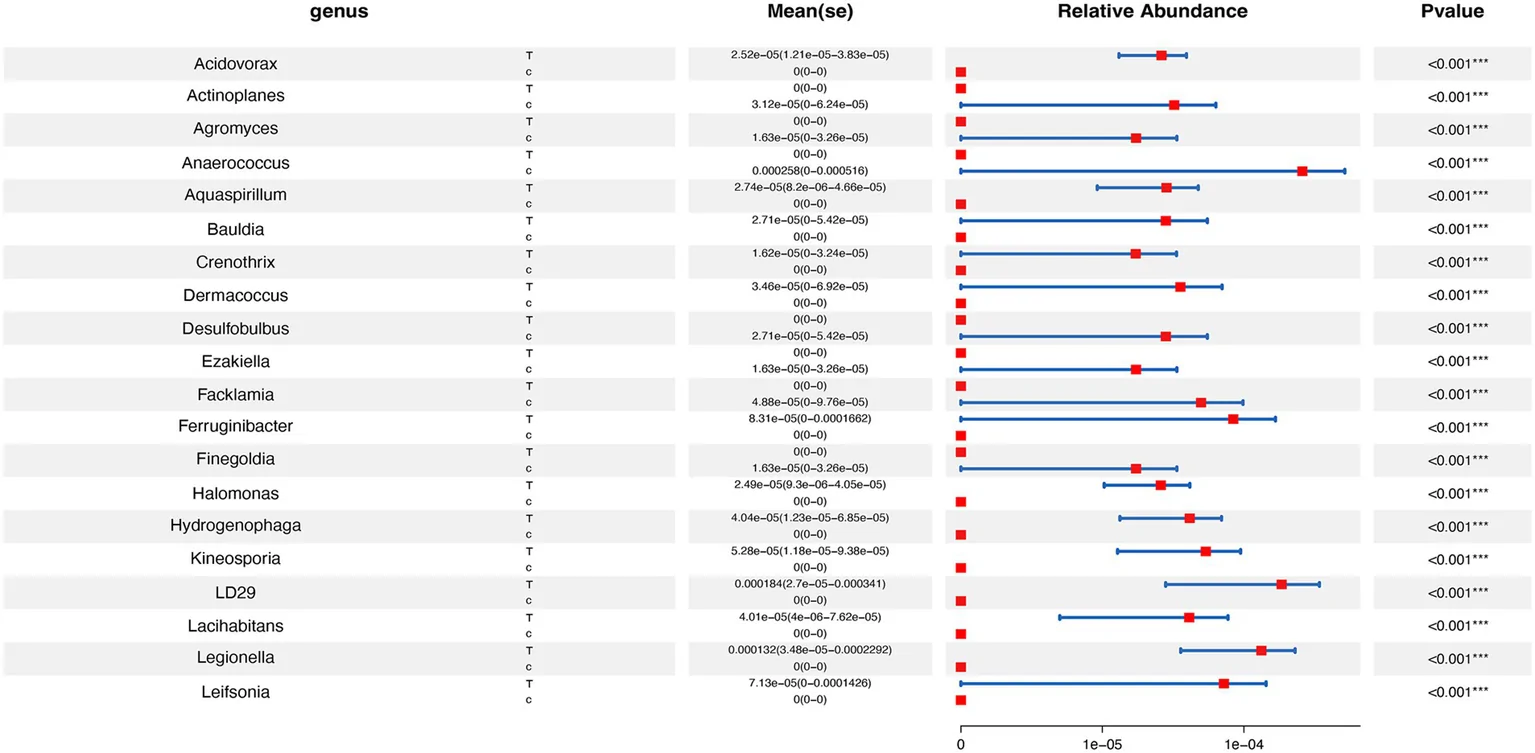
Metastats analysis of differentially abundant genera between the rice paddy (T) and pond (C) groups. Data are presented as mean relative abundance ± SE, with *p*-values indicating significance (***p < 0.001). The bar plot shows relative abundance comparisons between the two groups.

#### 3.5.4 PICRUSt2 functional prediction

PICRUSt2 analysis was performed as an exploratory prediction of the potential metabolic functions of the gut content-associated microbial communities based on 16S rRNA gene sequences. The relative abundances of predicted KEGG pathways at the second-level category across six taxonomic levels (phylum, class, order, family, genus, and species) are shown in Supplementary Figures S3–S8. At the genus level (Supplementary Figure S7), the predominant KEGG pathway categories in both groups were largely similar and included “Global and overview maps,” “Carbohydrate metabolism,” “Amino acid metabolism,” “Energy metabolism,” “Lipid metabolism,” “Membrane transport,” “Signal transduction,” “Replication and repair,” and “Translation.” These categories collectively constituted the majority of the predicted functional repertoire. However, STAMP analysis did not reveal a clear overall functional separation between the pond (C) and rice paddy (T) groups, and no significant difference was detected for the “Infectious diseases: Bacterial” pathway (Figure 12). Therefore, the PICRUSt2 results are interpreted with caution and are not used as the primary basis for functional inference. Functional comparisons between the two groups are instead based mainly on the differentially abundant taxa identified by LEfSe and Metastats.

**Figure 12:**
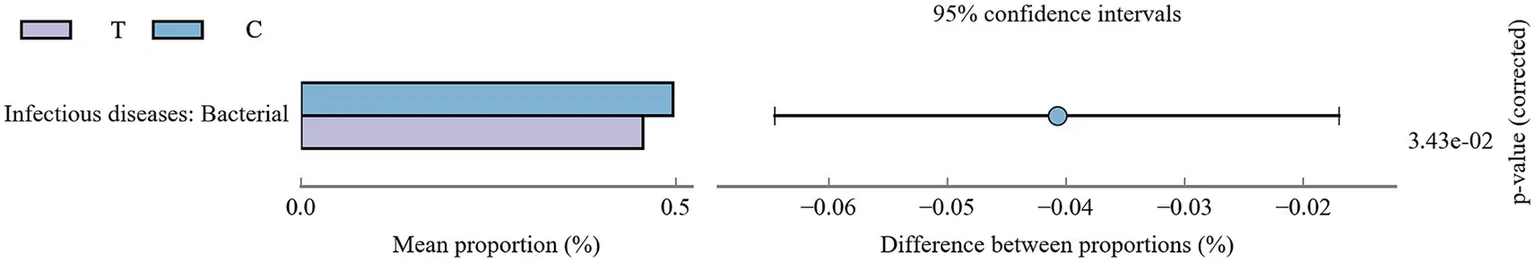
STAMP analysis of KEGG pathway differences between the rice paddy-cultured (T) and pond-cultured (C) groups. The left panel shows the mean proportion of each KEGG pathway in the two groups, the middle panel displays the 95% confidence intervals for the difference in proportions, and the right panel indicates the *p*-values.

STAMP analysis of KEGG pathways (Figure 12) showed that the category “Infectious diseases: Bacterial” was detected at negligible levels in both groups, with no significant difference between the rice paddy (T) and pond (C) groups.

BugBase phenotype prediction (Figure 13) was used as an exploratory, hypothesis-generating analysis to compare predicted microbial phenotypes between the two groups. The pond group (C) exhibited higher abundances of Stress_Tolerant, Potentially_Pathogenic, Gram_Negative, Forms_Biofilms, Facultatively_Anaerobic, and Contains_Mobile_Elements phenotypes compared to the rice paddy group (T). In contrast, the rice paddy group (T) exhibited higher abundances of Gram_Positive, Anaerobic, and Aerobic phenotypes. These results suggest that the two farming systems are associated with differential predicted microbial phenotypic profiles. The pond system was associated with a predicted phenotype profile characterized by higher stress tolerance, potential pathogenicity, Gram-negative characteristics, biofilm formation, facultative anaerobiosis, and mobile genetic elements, whereas the rice-crayfish co-culture system was associated with a predicted phenotype profile enriched in Gram-positive, anaerobic, and aerobic phenotypes. However, because BugBase is a predictive tool based on 16S rRNA gene data, and because the present dataset is based on intestinal lumen contents with no paired water or sediment microbiota data, these results should be interpreted with caution and are considered exploratory and hypothesis-generating only. They are not used as primary evidence for host health, disease status, or functional capability.

**Figure 13:**
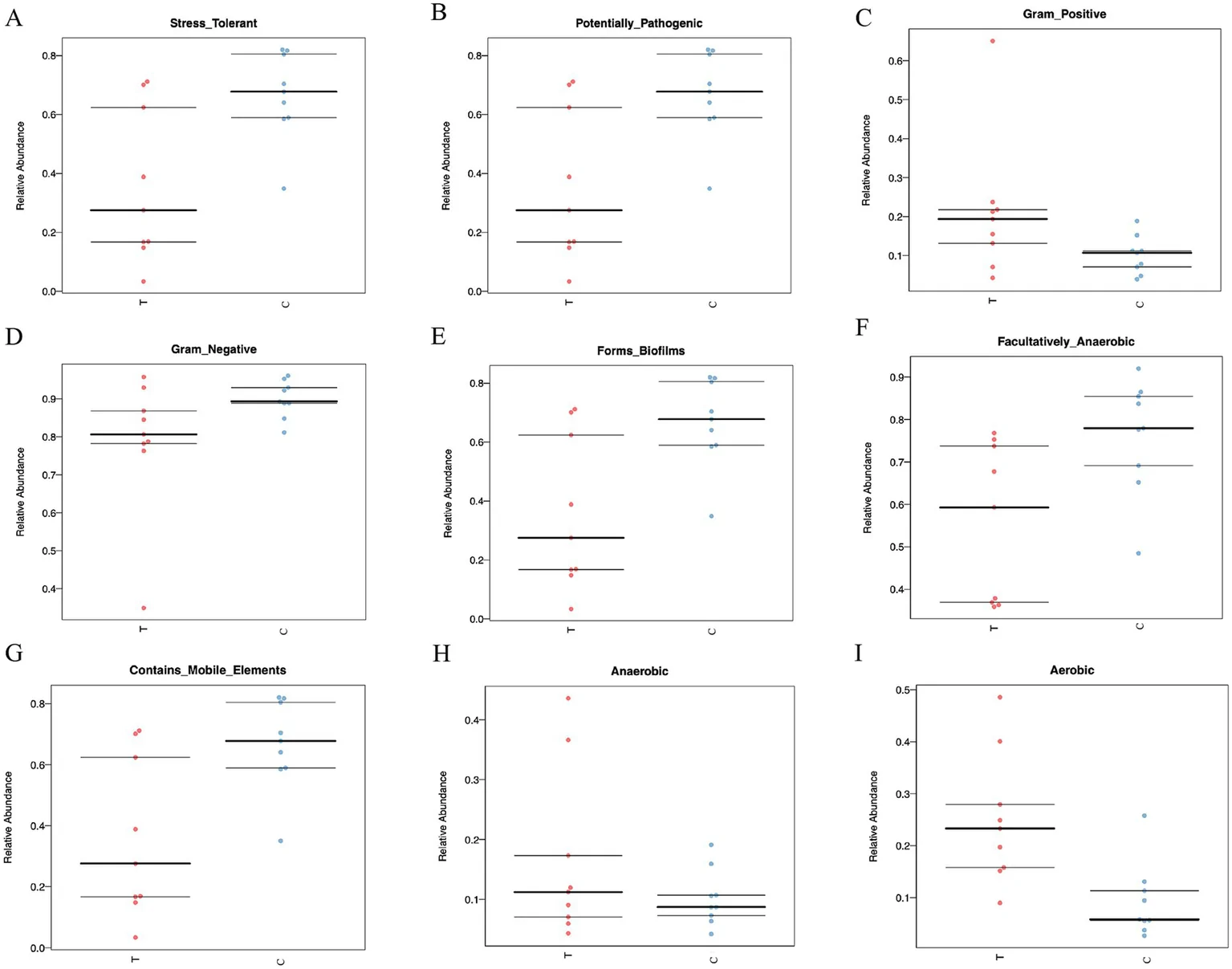
BugBase phenotype prediction of gut microbial communities in the rice paddy-cultured (T) and pond-cultured (C) groups. The *x*-axis represents the group names, and the y-axis represents the relative abundance (%) of each predicted phenotype. The three horizontal lines within each box indicate the lower quartile, median, and upper quartile, respectively. Significant differences between groups were observed for the following phenotypes: **(A)** Stress tolerant; **(B)** Potentially pathogenic; **(C)** Gram-positive; **(D)** Gram-negative; **(E)** Forms biofilms; **(F)** Facultatively anaerobic; **(G)** Contains mobile elements; **(H)** Anaerobic; **(I)** Aerobic. Colors distinguish the two groups as indicated in the legend.

## 4 Discussion

This study examined the intestinal physiological and microbial adaptations of *Procambarus clarkii* reared in intensive pond monoculture and integrated rice-crayfish co-culture. By combining intestinal enzyme assays with 16S rRNA sequencing of the gut content-associated microbiota, we found that the two farming systems are associated with coordinated, system-specific intestinal adaptation patterns. These findings extend our previous hepatopancreatic transcriptomic results (Gao et al., 2026) and suggest that the intestine plays an important role in mediating the trade-off between rapid biomass production and systemic resilience. Our results suggest that the rice paddy system is associated with an intestinal phenotype characterized by enhanced carbohydrate digestion, fortified antioxidant defenses, reduced oxidative damage, lower immune stimulation, and a more diverse and potentially beneficial lumen-associated microbiota. In contrast, the pond system is associated with a phenotype of high lipid/protein digestion, elevated oxidative stress, reduced immune barrier enzyme activities (compromised mucosal immune defense), higher energy transport workload, and a lumen-associated microbiota enriched with potentially opportunistic pathogens.

### 4.1 Dietary-driven digestive enzyme plasticity

The observed differences in intestinal digestive enzyme activities provide direct functional evidence of metabolic adaptation to the contrasting dietary compositions of the two farming systems. Crayfish in the rice paddy group exhibited significantly higher amylase activity, which aligns with the carbohydrate-rich nature of their natural diet (approximately 47.3% carbohydrates), comprising detritus, plankton, and plant-based materials (Gao et al., 2026). This elevation in amylase activity represents a classical example of substrate-induced enzyme modulation, a well-documented phenomenon in decapod crustaceans where digestive enzyme profiles closely mirror dietary composition (Figueiredo and Anderson, 2009; Pavasovic et al., 2004). Previous studies have demonstrated that dietary carbohydrate-to-lipid ratios significantly influence intestinal amylase activity in *P. clarkii*, with higher carbohydrate levels promoting increased amylase expression. The capacity to upregulate carbohydrate digestion is particularly advantageous in rice paddies, where plant-derived organic matter constitutes a major food source, enabling crayfish to efficiently exploit the available nutritional resources within the integrated agro-ecosystem. Furthermore, studies have shown that dietary modulation can significantly affect digestive enzyme profiles, with protease and amylase activities responding differentially to various feed types (Hu et al., 2026).

Conversely, pond-cultured crayfish displayed significantly higher lipase and total protease activities, corresponding to the high-fat (6.8%) and high-protein (32.5%) content of the formulated feed (Gao et al., 2026). This pattern suggests that the intensive pond system, with its nutrient-dense, energy-rich diet, is associated with a metabolic orientation towards lipid and protein utilization, supporting rapid somatic growth. The elevated lipase activity is particularly notable, as it facilitates the digestion of the high lipid content in the formulated feed, while the increased total protease activity reflects the enhanced capacity to process the substantial dietary protein load. These findings are consistent with previous studies on crustacean digestive physiology, which have demonstrated that dietary protein and lipid levels are primary determinants of corresponding digestive enzyme activities (Hu et al., 2026; Sarmiento Cárdenas et al., 2026). Research has also shown that digestive enzyme activities, including protease, lipase, and amylase, are significantly influenced by environmental factors such as temperature, with optimal temperatures promoting maximal enzyme activity (Zhou et al., 2021). Collectively, the digestive enzyme profiles suggest a fundamental metabolic divergence: the pond system is associated with a lipid/protein-centric anabolic state conducive to rapid biomass accumulation, whereas the rice paddy system is associated with carbohydrate utilization, reflecting adaptation to a more plant-based, detrital food web.

### 4.2 Systemic and intestinal antioxidant defense: a coordinated resilience phenotype

One of the most striking findings of this study is the marked enhancement of intestinal antioxidant capacity in rice paddy-cultured crayfish, evidenced by significantly higher activities of SOD, CAT, and GSH-Px, coupled with significantly lower MDA content. This intestinal “high antioxidant, low oxidative damage” phenotype is remarkably consistent with our previous observations in the hepatopancreas of the same experimental animals (Gao et al., 2026), suggesting that the enhanced antioxidant defense is not merely a local intestinal response but a systemic physiological trait associated with the rice-crayfish co-culture system. Such coordinated upregulation of antioxidant enzymes across multiple tissues suggests that the rice paddy environment may be associated with greater investment in cellular maintenance and redox homeostasis, potentially as an adaptive strategy to cope with the more variable and complex conditions of the integrated agro-ecosystem, including fluctuations in temperature, dissolved oxygen, and exposure to plant-derived allelochemicals (Guo et al., 2020; Luo et al., 2022). SOD, CAT, and GSH-Px are recognized as the principal antioxidant substances in crustaceans, working synergistically to scavenge reactive oxygen species and maintain cellular redox balance (Zheng et al., 2018; Xia et al., 2013).

In contrast, pond-cultured crayfish exhibited lower antioxidant enzyme activities and higher MDA levels in the intestine, mirroring the elevated oxidative damage previously observed in their hepatopancreas (Gao et al., 2026). The pond group also displayed elevated baseline cortisol-like immunoreactivity in hemolymph (Gao et al., 2026), suggesting a state of chronic physiological stress. This pattern indicates that the intensive pond environment, while associated with rapid growth, is associated with a significant oxidative cost. Environmental stressors such as pollutant exposure (CuSO₄, herbicides) and intensive rearing conditions have been shown to induce oxidative stress in *P. clarkii*, characterized by initial increases followed by declines in SOD, CAT, and GSH-Px activities, accompanied by elevated MDA levels (Wang et al., 2023). Similarly, low-temperature stress has been demonstrated to significantly affect antioxidant enzyme activities in the hepatopancreas and hemolymph of *P. clarkii*, with SOD and GSH-PX activities initially increasing then decreasing under prolonged stress (Ding et al., 2025a). The combination of high nutrient intake, intensive feeding, and potentially higher metabolic rates may lead to increased production of reactive oxygen species (ROS) that overwhelm the endogenous antioxidant capacity, resulting in lipid peroxidation and cellular damage (Valgas et al., 2024). This growth-stress trade-off is a recurrent theme in life-history theory, where investment in rapid somatic growth often occurs at the expense of maintenance and repair mechanisms (Stearns, 1992). The concordance between intestinal and hepatic antioxidant responses is consistent with the possibility that the rice-crayfish co-culture system is associated with a holistic, organism-wide stress-resistance-oriented syndrome, whereas the pond system is associated with a growth-oriented syndrome with elevated systemic oxidative burden.

### 4.3 Intestinal immune barrier and energy metabolism: contrasting physiological demands

The significantly higher activities of intestinal mucosal immune barrier enzymes (AKP and ACP) in the rice paddy group represent another key physiological distinction between the two systems. AKP and ACP are important components of the non-specific immune defense in the intestinal mucosa (Liu et al., 2024). The elevated activities observed in the rice paddy group likely reflect an enhanced mucosal immune barrier and a more robust non-specific immune status, consistent with the stress-resistance-oriented phenotype observed in this system. In contrast, the lower AKP and ACP activities in the pond group may indicate that intensive pond culture suppresses or exhausts intestinal mucosal immune defense, or that the mucosal barrier is compromised under high-density, high-input conditions. This interpretation is consistent with the enrichment of potentially pathogenic taxa and the higher abundance of ‘Potentially pathogenic’ phenotypes predicted by BugBase in the pond group.

Concurrently, the pond group exhibited significantly higher intestinal Na^+^/K^+^-ATPase activity, suggesting enhanced epithelial energy metabolism and active ion transport. Na^+^/K^+^-ATPase is the primary driving force for nutrient absorption in the intestinal epithelium, maintaining the electrochemical gradient necessary for the cotransport of glucose, amino acids, and other nutrients (Skou, 1998). The elevated activity in the pond group is consistent with the higher absorptive workload associated with the nutrient-dense formulated diet, which requires intensive transport processes to assimilate the substantial dietary protein, lipid, and carbohydrate load. This is consistent with the growth-oriented phenotype of pond-cultured crayfish, where the intestinal epithelium is continuously engaged in high-capacity nutrient uptake to support rapid biomass accumulation. In the rice paddy group, the lower Na^+^/K^+^-ATPase activity reflects a reduced intestinal transport workload, commensurate with the lower nutrient density and availability of the natural diet. This metabolic adjustment conserves energy that can be allocated to other maintenance functions, such as antioxidant defense and immune surveillance, further supporting the stress-resistance-oriented syndrome.

### 4.4 Gut content-associated microbial community: a mirror of environmental and physiological conditions

The 16S rRNA sequencing analysis showed profound differences in the gut content-associated microbial community structure between the two farming systems, providing a microbial dimension to the observed physiological divergence. The rice paddy group exhibited significantly higher alpha diversity (Shannon and Chao1 indices), suggesting a more diverse and even lumen-associated microbiota. This is consistent with the general principle that environmental complexity and dietary diversity promote higher microbial diversity (Yatsunenko et al., 2012), which is often associated with enhanced host health, resilience, and metabolic flexibility (Sommer and Bäckhed, 2013). Studies on *P. clarkii* gut content-associated microbiota under different aquaculture conditions have demonstrated that rearing environments significantly shape microbial community composition and diversity, with integrated systems often supporting more diverse microbial communities (Shui et al., 2020). The rice paddy environment, with its diverse natural food sources and structurally complex habitat, may provide a richer array of substrates and ecological niches for microbial colonization, which may in turn be associated with a more diverse gut ecosystem. However, because water microbiota was not sampled, this possibility remains speculative.

At the phylum level, the rice paddy group showed a higher relative abundance of *Bacteroidota*, while the pond group was enriched in *Proteobacteria*. This compositional shift is ecologically and physiologically significant. *Bacteroidota* are renowned for their capacity to degrade complex polysaccharides, including plant-derived cellulose, hemicellulose, and pectin (Thomas et al., 2011). The enrichment of *Bacteroidota* in the rice paddy group aligns with the carbohydrate-rich, plant-based diet of these crayfish and may contribute to the efficient fermentation and utilization of dietary fibers. Previous research has demonstrated that fermented feed significantly increases *Bacteroidetes* abundance while decreasing *Proteobacteria* in adult *P. clarkii*, suggesting that dietary composition is a key determinant of phylum-level microbial shifts (Zhang et al., 2020). In contrast, *Pseudomonadota* is a phylum that includes many opportunistic pathogens, and its enrichment has been associated with gut dysbiosis and disease susceptibility in various aquatic species (Shin et al., 2015). The higher abundance of *Pseudomonadota* in the pond group may be associated with the intensive feeding and high stocking density of the pond system, although the relative contribution of water microbiota to this pattern could not be assessed in the present study. This pattern is consistent with the elevated AKP and ACP activities observed in the pond group, suggesting that the lumen-associated microbiota in the pond system may exert a higher immune-stimulatory pressure on the host.

The genus-level and LEfSe analyses further refined these community differences. The rice paddy group was characterized by enrichment of *Bacteroides* and *Candidatus_Bacilloplasma*, taxa commonly associated with the degradation of complex carbohydrates and maintenance of gut homeostasis (Wexler, 2007). In contrast, the pond group was enriched in *Citrobacter*, *Acinetobacter*, and other members of the orders *Enterobacterales* and *Pseudomonadales*. Many species within these genera, such as *Citrobacter freundii* and *Acinetobacter* spp., are known opportunistic pathogens in aquatic animals, capable of causing disease under conditions of host stress or immunosuppression (Austin and Austin, 2007). *Citrobacter freundii* has been identified as a common pathogen in commercially available *P. clarkii*, capable of causing significant health impacts (Huang et al., 2021). The enrichment of these taxa in the pond group is noteworthy, as it suggests that the intensive pond system may be associated with the proliferation of potentially pathogenic bacteria in the crayfish gut. This pattern may be associated with the higher nutrient load, elevated stress levels, and potentially compromised host immunity in pond-cultured crayfish, which could create a permissive environment for opportunistic pathogens. However, without water microbiota data, the possibility of environmental microbial transfer cannot be distinguished from host-related factors. Microplastic exposure has also been shown to increase the abundance of opportunistic genera such as *Acinetobacter* in *P. clarkii* intestines, further supporting the link between environmental stressors and pathogenic microbial enrichment (Guillén-Watson et al., 2023). The BugBase phenotype prediction was considered exploratory and hypothesis-generating. Although the pond group showed a higher abundance of “Potentially pathogenic” phenotypes, this result should be interpreted cautiously because BugBase is predictive and water/sediment microbiota were not characterized. Therefore, BugBase results are not used as primary evidence for host health or disease status.

PICRUSt2 is a predictive approach, and its results should be regarded as exploratory. Because no clear overall functional separation was detected, we base our functional discussion on the biomarker taxa identified by LEfSe and Metastats. The enrichment of *Bacteroides* and *Candidatus_Bacilloplasma* in the rice paddy group is consistent with a potential capacity for degrading complex carbohydrates, whereas the enrichment of *Citrobacter* and *Acinetobacter* in the pond group is consistent with the presence of taxa that include opportunistic pathogens. These taxonomic differences provide a more reliable basis for functional inference than PICRUSt2 predictions alone.

Limitations: Because 16S rRNA sequencing was performed on intestinal lumen contents rather than on mucosal tissue or mucosal scrapings, the present dataset cannot distinguish true resident or symbiotic microbes from transient microbes in the lumen. In addition, water and sediment samples from the pond and rice paddy systems were not collected. Therefore, the contribution of environmental microbiota to the composition of the gut content-associated microbiota of *P. clarkii* could not be directly evaluated. Accordingly, the observed differences in gut content-associated microbial communities between the two groups should be interpreted as associations with the respective farming systems, rather than as evidence of a causal transfer from water or sediment microbiota. Future studies incorporating paired water, sediment, gut content, and mucosal tissue samples, together with absolute quantification, metagenomics, or cultivation-based approaches, are needed to clarify the extent to which environmental microbial communities shape the true resident or symbiotic gut microbiota of crayfish under different aquaculture systems.

### 4.5 Integrating intestinal and hepatopancreatic adaptations: a multi-organ perspective on environmental adaptation

The integration of the present intestinal findings with our previous hepatopancreatic transcriptomic results (Gao et al., 2026) provides a comprehensive, multi-organ perspective on how *P. clarkii* adapts to contrasting aquaculture environments. The hepatopancreas, as the central organ for metabolism, detoxification, and nutrient storage, exhibited a “growth-oriented” transcriptional profile in the pond group, characterized by upregulated pathways for lipid biosynthesis and anabolic metabolism, but also elevated markers of oxidative stress and endocrine load. In contrast, the hepatopancreas of rice paddy-crayfish showed enhanced expression of pathways related to detoxification, xenobiotic metabolism, and endocrine regulation (upregulation of *SULT1E1*), reflecting a metabolic state geared toward homeostasis and resilience (Gao et al., 2026).

The present study suggests that the intestine mirrors and complements these hepatic adaptations. The intestinal digestive enzyme profiles directly reflect the dietary substrate availability in each system, while the antioxidant, immune barrier, and energy metabolism enzymes reveal the local physiological costs and demands imposed by each environment. The gut content-associated microbial community adds another layer of complexity, acting as a “metabolic organ” that interacts with the host’s intestinal physiology and contributes to the overall adaptive phenotype. Research on the intestinal immune response of *P. clarkii* to acute heat stress has shown that oxidative stress parameters, antioxidant enzyme activities, and immunity-related enzymes (AKP, ACP, lysozyme) in the intestine all respond dynamically to environmental challenges, with parameters tending to increase, peak, and then decrease over time (Guo et al., 2020). Similarly, studies on the intestinal lumen-associated microbiota of *P. clarkii* in integrated crayfish-rice cultivation models have demonstrated that microbial community composition is closely correlated with host health status and can be significantly affected by rearing conditions (Shui et al., 2020). The concordance between the intestinal and hepatic responses—both showing enhanced antioxidant capacity and reduced oxidative damage in the rice paddy group, and elevated metabolic load and stress markers in the pond group—suggests that the two systems are associated with coherent, organism-wide patterns. The rice-crayfish co-culture system is associated with a stress-resistance-oriented syndrome characterized by systemic antioxidant enhancement, efficient carbohydrate utilization, enhanced mucosal immune barrier function (higher AKP and ACP activities), and a diverse, potentially beneficial lumen-associated microbiota. Conversely, the intensive pond system is associated with a growth-oriented syndrome characterized by high lipid/protein anabolism, elevated physiological load, chronic stress, compromised mucosal immune defense (lower AKP and ACP activities), and a lumen-associated microbiota enriched in opportunistic pathogens.

This multi-organ integration is critical for understanding the biological trade-offs inherent to different aquaculture production models. The pond system successfully achieves its primary objective of rapid biomass production, but this comes at the cost of systemic physiological burden, oxidative damage, compromised intestinal mucosal immune defense, and potentially increased susceptibility to disease. The rice-crayfish co-culture system, while producing smaller individuals, yields animals with superior health indicators, enhanced resilience, and a more balanced host–microbe interaction. This trade-off between productivity and robustness is a fundamental consideration for sustainable aquaculture development, where the choice of production system may depend on whether the priority is maximum yield or product quality, system resilience, and ecosystem services (Naylor et al., 2021; Zhu et al., 2024).

### 4.6 Implications for aquaculture practice and sustainability

The findings of this study have significant implications for the optimization of crayfish aquaculture practices. For intensive pond systems, our results highlight a potential conflict between maximizing growth and maintaining animal health and welfare. The observed “growth-oriented syndrome” characterized by elevated oxidative stress, reduced immune barrier enzyme activities (compromised mucosal immune defense), and enrichment of potentially pathogenic bacteria in gut contents, suggests that current intensive management practices may inadvertently compromise the long-term health and disease resistance of the stock. To mitigate these risks, several strategies could be considered:

1. Optimized stocking density: Reducing stocking densities could alleviate stress and reduce the microbial challenge load, potentially lowering the immune stimulation and oxidative burden observed in pond-cultured crayfish (Koretskyi and Kononenko, 2024).
2. For intensive pond systems, our results showed elevated oxidative stress (higher MDA, lower SOD/CAT/GSH-Px), compromised mucosal immune defense (lower AKP/ACP activities), and enrichment of potentially pathogenic bacteria (e.g., Citrobacter, Acinetobacter) together with a higher relative abundance of Proteobacteria. Therefore, we now suggest that dietary supplementation with prebiotics (e.g., inulin) or probiotics could be a targeted strategy to mitigate these specific issues: enhancing intestinal antioxidant capacity, restoring mucosal immune enzyme activities (AKP, ACP), and potentially supporting a more balanced gut microbiota by suppressing opportunistic pathogens and increasing beneficial taxa such as *Bacteroidota.* This is directly supported by studies in *P. clarkii* showing that inulin improves digestive enzyme activities, intestinal morphology, and non-specific immune enzymes (Wang et al., 2024), and by evidence that probiotics enhance antioxidant capacity and immune response in crustaceans (Zhang et al., 2025).

For integrated rice-crayfish systems, our results indicate that this system already supports higher antioxidant defense, enhanced mucosal immune barrier function, and a more diverse lumen-associated microbiota enriched in potentially beneficial taxa. Therefore, we suggest that the priority should be to preserve these natural advantages by maintaining the low-input, natural food web and habitat complexity, rather than relying on excessive feed additives.

1. Environmental enrichment: Introducing structural complexity, such as submerged aquatic plants or artificial shelters, could reduce stress and aggression, while potentially supporting a more diverse and potentially beneficial gut microbial community (Baird et al., 2006).

For integrated rice-crayfish systems, this research provides physiological and microbial validation of their ecological and health benefits. The “stress-resistance-oriented syndrome” suggests that these systems may be associated with superior health indicators, enhanced resilience, and a more robust lumen-associated microbiota. This supports the continued promotion of rice-crayfish co-culture as a sustainable aquaculture practice that not only reduces reliance on external feed inputs and chemical inputs but may also be associated with healthier and potentially more resilient animals (Zhang et al., 2023; Li et al., 2024). The reduced oxidative damage and lower immune stimulation in the rice paddy group suggest that the integrated environment provides a more balanced physiological state, which could translate to higher survival rates under disease challenges or environmental perturbations (Dong et al., 2024). Furthermore, the ecosystem services provided by rice-crayfish co-culture, such as pest control, nutrient recycling, and improved water quality, contribute to the overall sustainability of the agro-ecosystem (Ahmed and Garnett, 2011; Turcios and Papenbrock, 2014).

## 5 Conclusion

In conclusion, this descriptive study suggests that intensive pond monoculture and integrated rice-crayfish co-culture systems are associated with distinct intestinal physiological and microbial patterns in *Procambarus clarkii*. The rice paddy system was associated with a more diverse gut content-associated microbial community, whereas the pond system was associated with enrichment of Pseudomonadota and taxa that include opportunistic pathogens. These patterns should be interpreted as associations between farming systems and lumen-associated microbial communities, rather than as evidence for differences in true resident symbiotic microbiota.

## Data Availability

The raw 16S rRNA gene sequencing data generated in this study have been deposited in the Mendeley Data repository and are available at https://data.mendeley.com/datasets/9f4t8k622p/1 (DOI: 10.17632/9f4t8k622p.1). Further inquiries can be directed to the corresponding author(s).

## Footnotes

1
www.majorbio.com
