# Supplementary material for: Intestinal adaptation of crayfish to pond and rice paddy farming: a microbiome-enzyme perspective
Source: Front Microbiol. 2026 Oct 26;17:1989196. doi: 10.3389/fmicb.2026.1989196 (PMC13745700; doi:10.3389/fmicb.2026.1989196)
Supplement: Supplementary file 1 [file Supplementary_file_1.docx]

**Supplementary Tables and Figures**

**Table S1.** Summary of sequencing data processing statistics for each sample.

| Sample ID | Raw Reads | Clean Reads | Denoised Reads | Merged Reads | Non-chimeric Reads |
| --- | --- | --- | --- | --- | --- |
| C11 | 80,225 | 66,333 | 66,260 | 64,283 | 59,170 |
| C12 | 80,006 | 68,974 | 68,867 | 67,330 | 65,698 |
| C13 | 79,720 | 66,568 | 66,440 | 64,587 | 61,772 |
| C21 | 79,893 | 67,440 | 67,398 | 66,611 | 62,710 |
| C22 | 80,021 | 65,687 | 65,624 | 64,912 | 63,413 |
| C23 | 80,057 | 67,337 | 67,205 | 65,998 | 64,356 |
| C31 | 79,757 | 67,094 | 66,954 | 63,967 | 56,086 |
| C32 | 79,970 | 67,425 | 67,276 | 64,582 | 58,991 |
| C33 | 79,657 | 66,800 | 66,687 | 64,451 | 58,299 |
| C41 | 80,127 | 67,898 | 67,798 | 65,695 | 61,223 |
| C42 | 79,943 | 68,393 | 68,339 | 66,550 | 61,682 |
| C43 | 79,800 | 66,930 | 66,832 | 64,901 | 61,634 |
| C51 | 79,873 | 67,276 | 67,194 | 65,120 | 59,462 |
| C52 | 79,834 | 66,692 | 66,649 | 65,620 | 61,370 |
| C53 | 80,315 | 67,753 | 67,626 | 64,887 | 60,863 |
| C61 | 80,016 | 66,020 | 65,944 | 63,581 | 57,244 |
| C62 | 80,179 | 66,123 | 66,063 | 64,149 | 58,303 |
| C63 | 80,044 | 66,788 | 66,630 | 64,181 | 59,194 |

Note: Sample ID, sample name; Raw Reads, number of raw paired-end reads; Clean Reads, number of high-quality reads after quality filtering; Denoised Reads, number of reads after DADA2 denoising; Merged Reads, number of reads merged from paired-end reads; Non-chimeric Reads, number of final high-quality reads after chimera removal.

**Table S2.** Number of features (ASVs) and sequences identified in each sample.

| Sample | OUT_Num | Seqs_Num |
| --- | --- | --- |
| C11 | 246 | 58,978 |
| C12 | 463 | 65,485 |
| C13 | 359 | 61,611 |
| C21 | 175 | 62,627 |
| C22 | 267 | 63,316 |
| C23 | 450 | 64,210 |
| C31 | 417 | 55,838 |
| C32 | 383 | 58,765 |
| C33 | 347 | 58,123 |
| C41 | 315 | 61,041 |
| C42 | 279 | 61,499 |
| C43 | 365 | 61,478 |
| C51 | 273 | 59,309 |
| C52 | 224 | 61,269 |
| C53 | 391 | 60,625 |
| C61 | 262 | 57,098 |
| C62 | 267 | 58,176 |
| C63 | 369 | 59,020 |
| Total | 3,321 | 1,088,468 |

Note: Sample, sample name; Feature_Num, number of amplicon sequence variants (ASVs) obtained after DADA2 denoising; Seqs_Num, total number of sequences assigned to the corresponding features. ASVs represent the true biological sequences distinguished at single-nucleotide resolution. “Total” indicates the sum of features across all samples (not the sum of per-sample features, as features may be shared) and the total number of sequences from all samples after chimera removal.

**Figure S1.** Rarefaction curves of the 16S rRNA gene sequencing libraries for all samples.

**Figure S2.** Shannon diversity index curves of the 16S rRNA gene sequencing libraries for all samples.

**Figure S3. KEGG metabolic pathway relative abundance profiles at the phylum level predicted by PICRUSt2.**

**Figure S4. KEGG metabolic pathway relative abundance profiles at the class level predicted by PICRUSt2.**

**Figure S5. KEGG metabolic pathway relative abundance profiles at the order level predicted by PICRUSt2.**

**Figure S6. KEGG metabolic pathway relative abundance profiles at the family level predicted by PICRUSt2.**

**Figure S7. KEGG metabolic pathway relative abundance profiles at the genus level predicted by PICRUSt2.**

**Figure S8. KEGG metabolic pathway relative abundance profiles at the species level predicted by PICRUSt2.**
